# Mutual Impact of Dietary Antioxidants and *TNF-α* rs1800629 on Insulin Levels in Adults with Obesity

**DOI:** 10.3390/nu17142345

**Published:** 2025-07-17

**Authors:** Erika Sierra-Ruelas, Barbara Vizmanos, Juan José López Gómez, Daniel Rico, J. Alfredo Martínez, Daniel A. De Luis

**Affiliations:** 1 Doctorados en Cs. de la Nutrición Traslacional y de la Salud Pública, Laboratorio Evaluación Estado Nutricio, Instituto de Nutrigenética y Nutrigenómica Traslacional, Centro Universitario de Ciencias de la Salud, Universidad de Guadalajara, Guadalajara 44340, Jalisco, Mexico; ln.erikasierra@outlook.com (E.S.-R.); bvizmanos@yahoo.com.mx (B.V.); 2 Dirección de Investigación, Secretaría de Salud Jalisco, Guadalajara 44100, Jalisco, Mexico; 3 Centro de Investigación de Endocrinología y Nutrición Clínica, Universidad de Valladolid, 47002 Valladolid, Spain; jlopezgo@saludcastillayleon.es (J.J.L.G.); daniel.rico@uva.es (D.R.); 4 Health Research Institute of Valladolid (IBioVALL), 47010 Valladolid, Spain; 5 Nutrición de Precisión y Programa de Salud Cardio Metabólica, Instituto Madrileño de Estudios Avanzados (IMDEA), Consejo Superior de Investigaciones Científicas-Campus de Excelencia Internacional (CSIC-CEI), 28049 Madrid, Spain; 6 Centro de Investigación Biomédica en Red Fisiopatologia de la Obesidad y la Nutrición (CIBEROBN), Instituto de Salud Carlos III, 28029 Madrid, Spain

**Keywords:** *TNF-α*, insulin, CDAI, antioxidants, obesity, gene-diet interaction

## Abstract

**Background/objectives:** The interplay between genetic factors and nutritional patterns is critical in understanding metabolic health. This analysis evaluated the potential reciprocal relationships between the *TNF-α* -308 G/A gene polymorphism, the Composite Dietary Antioxidant Index (CDAI), and insulin-related variables in Spanish adults with obesity. **Methods:** A cross-sectional analysis was conducted in 292 adults with obesity. Anthropometric, biochemical, and dietary variables were assessed. *TNF-α* -308 G/A genotyping was performed. Associations and potential interactions between CDAI and genotype on insulin and homeostatic model assessment for insulin resistance (HOMA-IR) were examined using multivariate regression and two-way ANOVA. **Results:** Higher CDAI scores were significantly associated with lower insulin levels (*p* < 0.001) and HOMA-IR (*p* < 0.001), regardless of genotype. Carriers of the A allele (GA/AA) showed a non-significant trend toward higher insulin levels (*p* = 0.087) and a steeper decrease in insulin levels with increasing CDAI, with a significant interaction observed between *TNF-α* genotype and CDAI (interaction *p* = 0.003). Multivariate analyses confirmed that CDAI and *TNF-α* genotype were independently associated with insulin and HOMA-IR levels. However, interaction terms were not consistently significant across all models. **Conclusions:** These findings emphasize the potential of antioxidant-rich diets to help modulate the influence of pro-inflammatory genotypes on insulin resistance, highlighting the relevance of integrating genetic and dietary factors in managing obesity-related metabolic risks. Further studies are warranted to confirm these preliminary findings and to better understand the mechanisms underlying gene–diet interactions in metabolic regulation.

## 1. Introduction

Obesity is a critical public health burden, with rising prevalence affecting nearly all populations worldwide [1]. The exposome, which encompasses environmental exposures throughout the lifespan, play a significant role in obesity development, particularly through factors such as dietary habits and sedentarism, where genetic interactions must also be considered [2]. Notably, the pathways of obesity development also include mechanisms associated with chronic inflammation and oxidative stress [3,4]. These inflammatory and oxidative processes may contribute to metabolic dysregulation and are increasingly recognized as key components in the multifactorial causes of obesity [5]. Indeed, elevated levels of reactive oxygen species (ROS) have been linked to hyperglycemia, inflammation, and dyslipidemia [3]. Moreover, ROS may contribute to diseases such as type 2 diabetes by activating alternative downstream signaling pathways that are critically involved in insulin resistance and impaired insulin secretion [6].

In this context, insulin is a critical regulator of blood glucose levels, vasodilation, cell growth, and protein metabolism [7]. A decrease in the responsiveness of peripheral target tissues to insulin leads to glucose intolerance, a complex pathophysiological condition characterized by reduced insulin sensitivity, the impaired inhibition of hepatic glucose production, and the diminished stimulation of peripheral glucose uptake [6]. This condition is often accompanied by hyperinsulinemia, which attempts to help maintain glycemic homeostasis. Consequently, insulin resistance plays a pivotal role in the development and progression of metabolic diseases, including type 2 diabetes mellitus, hypertension, tumors, and nonalcoholic fatty liver disease, thereby establishing a common framework for understanding these chronic clinical features, where nutrient intake plays a significant role [7].

Some studies have shown that a diet rich in antioxidants, which reduce ROS levels, can improve glucose metabolism regulation, promote insulin secretion, and decrease insulin resistance [8,9]. Thus, dietary modifications may offer a valid approach to mitigate these metabolic disturbances [10]. In order to assess the impact of diet on inflammation and oxidative stress, the Composite Dietary Antioxidant Index (CDAI) has been developed. This index, calculated by considering the inflammatory and antioxidant properties of dietary nutrients, serves as a valuable tool for evaluating the influence of dietary patterns on health [11]. Notably, CDAI has been associated with elevated levels of tumor necrosis factor-α and interleukin-1β [12], and an even closer relationship with hypertension [13,14], type 2 diabetes mellitus [15,16], and cardiovascular disease has been reported [17,18]. A number of research findings indicate that certain antioxidants, including flavonoids [19], vitamin C [20], and carotenoids [21], are inversely associated with insulin resistance and type 2 diabetes mellitus risk. Additionally, selenium intake has been associated with favorable glycemic markers, such as lower HbA1c and insulin levels, indicating its potential role in glucose metabolism and type 2 diabetes management [22].

In addition to dietary factors, environmental, biological, and genetic influences also play pivotal roles in the complex regulation of glucose metabolism [23]. Among the genetic factors, specific genes involved in inflammatory and oxidative processes, such as tumor necrosis factor-alpha (*TNF-α*), have been implicated in the development of obesity and insulin resistance [24]. Thus, several single nucleotide variants (SNVs) have been identified within the promoter region of *TNF-α* gene, where the most extensively studied and well-characterized is the—-308 G/A (rs1800629) [25]. Regarding this *TNF-α* polymorphism, studies on potential associations with alterations in glycemic control and insulin levels have shown diverse results [26,27,28].

The *TNF-α* gene encodes a pro-inflammatory cytokine involved in the pathogenesis of insulin resistance and obesity [29]. The -308 G/A polymorphism, located in its promoter region, is associated with increased *TNF-α* expression, particularly in A allele carriers, due to its stronger transcriptional activity [30]. *TNF-α* impairs insulin signaling by inhibiting the tyrosine phosphorylation of IRS-1 and promoting its phosphorylation at serine 307, which disrupts downstream pathways, reduces GLUT4 translocation, and impairs glucose uptake [31,32]. It also increases lipolysis and free fatty acid concentrations, contributing to insulin resistance, while inducing oxidative stress and endothelial dysfunction through superoxide production [33,34]. In this context, other genes such as *PPAR-γ*, *IRS-1*, *AMPK*, *KCNJ11*, *FABP*, and *TCF7L2* also play roles in glucose metabolism and insulin sensitivity, influencing β-cell function, fatty acid handling, or intracellular signaling [35,36,37,38,39,40]. These genes form part of a complex regulatory network, highlighting the importance of gene–diet interactions in the development of insulin resistance and related metabolic disturbances. The frequency of the A allele of the *TNF-α* -308 G/A polymorphism varies among populations, ranging from approximately 5.3% in South Asians and 5.8% in East Asians to 13.4% in Europeans and 11.9% in Africans, with a global frequency of around 9%, according to data from the 1000 Genomes Project [41].

Since the discovery that the -308 G/A variant enhances *TNF-α* transcriptional activity, numerous studies have explored its association with various physiological and pathological conditions, including cancer [42,43], pulmonary diseases [44,45], type 2 diabetes [26,27], components of metabolic syndrome [25], and non-alcoholic fatty liver disease [46]. Some evidence suggests that this variant contributes to the development of obesity and insulin resistance; however, findings across studies remain inconsistent [28,47]. While certain investigations have reported a clear association between the A allele and increased insulin resistance or impaired glucose tolerance [30], others have failed to replicate these results [48,49], underscoring the need for further research to clarify its role in metabolic health.

We hypothesized that *TNF-α* -308 G/A polymorphism influences insulin levels and HOMA-IR in adults with obesity, and that this effect may be modified by dietary antioxidant intake. To the best of our knowledge, no previous studies have examined the combined influence of the *TNF-α* variants and the dietary antioxidant intake on circulating insulin levels. Therefore, this study aimed to evaluate the potential impact between the interaction of *TNF-α* rs1800629 variant and the CDAI on circulating insulin levels and HOMAR-IR in a Spanish adult population with obesity in order to determine a potential effect modification of antioxidant intake on insulinemia, depending on the genetic make-up.

## 2. Materials and Methods

### 2.1. The Study Population

In this cross-sectional study, a total number of 292 Spanish adults were included. Participants were recruited in 2007 and 2008 in Castilla y León, Spain. The recruitment of subjects was a non-probabilistic method of sampling. Patients were referred by Primary Care physicians to the Units of Nutrition of each Health Area in the Castilla y León autonomous community to evaluate their obesity status. The sample included adults, women (*n* = 187) and men (*n* = 105) with obesity (BMI > 30 kg/m^2^). Exclusion criteria included the history of cardiovascular disease or stroke within the previous 36 months, as well as the use of sulfonylurea, metformin, thiazolidinediones, insulin, glucocorticoids, antineoplastic agents, angiotensin receptor blockers, angiotensin-converting enzyme inhibitors, and psychoactive medications, attempting to overcome potential pharmacological interactions. Ethical approval was obtained from the Ethical Committee of Hospital Río Hortega with reference pi7 542, and a written informed consent was voluntarily signed by all participants prior to their participation. The study was performed in accordance with the principles of the Declaration of Helsinki regarding research involving humans.

### 2.2. Dietary Intake and CDAI

All enrolled subjects received nutritional instruction, incorporating the use of food scales and models to enhance portion size accuracy, to record their daily dietary intake in a 2-day food diary, including a weekend day [50]. Diary records were reviewed by a registered dietician and analyzed with a computer-based data evaluation system. National composition food tables were used as dietetical reference [51].

From the version of the CDAI developed by Wright et al. [11] a modification was incorporated, as in other similar studies [52,53], by including eight nutrients with potential antioxidant activity for study: vitamins A, C, and E; zinc; selenium; copper; fiber; and omega 3. The inclusion of fiber and omega-3 was based on their well-documented indirect antioxidant effects. Omega-3 fatty acids promote endogenous antioxidant enzyme activity and modulate inflammation-related transcription factors such as NF-κB and Nrf2. Dietary fiber contributes through its fermentation into short-chain fatty acids like butyrate, which support antioxidant defenses, and by enhancing the bioaccessibility of polyphenols and other phytochemicals present in fiber-rich foods [54,55]. The CDAI is the sum of the eight standardized dietary intake values of antioxidants, and was calculated with the following formula:

$$CDAI=\sum_{i=1}^{n=8}\left(\frac{individual\;intake-mean}{standard\;deviation}\right)$$

### 2.3. Anthropometric and Body Composition Measurements

Anthropometric and body composition measurements were performed following the established protocols [56] with participants wearing light clothing. Body weight was measured to an accuracy of 0.1 kg using a height measurement scale (Omrom Healthcare Inc., Kyoto, Japan), and BMI was computed as body weight/(height^2^) [57]. Waist circumference (measured at the narrowest point between the xiphoid process and the iliac crest) and hip circumference (measured at the widest point over the greater trochanters) were determined using a standard tape measure (OmronHealthcare Inc., Kyoto, Japan). The waist-to-hip ratio was subsequently calculated. Tetrapolar body electrical bioimpedance was used to determine body composition [58]. An electric current of 0.8 mA and 50 kHz was produced by a calibrated signal generator (Model 310e, Biodynamics Corporation, Seattle, WA, USA) and applied to the skin using adhesive electrodes placed on right-side limbs following standardized protocols [56]. The fat mass and lean mass were determined using resistance and reactance, which were then used to calculate the fat mass-to-lean mass ratio.

### 2.4. Biochemical Analysis and Clinical Data

Blood samples were obtained under fasting conditions. Serum total cholesterol (TC) and triglyceride (TG) concentrations were determined by enzymatic colorimetric assay (Technicon Instruments Ltd., Tarrytown, NY, USA). High-density lipoprotein cholesterol (HDL-c) was determined enzymatically in the supernatant after the precipitation of other lipoproteins with dextran sulfate–magnesium. Low-density lipoprotein cholesterol (LDL-c) was calculated using the Friedewald formula [59]. The TG and glucose index (TyG) was calculated as follows: Ln[fasting TG (mg/dL) × fasting glucose (mg/dL)]/2 [60].

Plasma glucose levels were determined using an automated glucose oxidase method (Glucose Analyzer 2, Beckman Instruments, Fullerton, CA, USA). Insulin was measured by enzymatic colorimetry (Insulin assay, WAKO Pure-Chemical Industries, Osaka, Japan) and HOMA-IR was calculated as follows: (fasting insulin (μU/mL) × fasting glucose (mg/dL)/405) [61]. C-reactive protein (CRP) was measured using immunoturbimetry (Roche Diagnostics GmbH, Mannheim, Baden-Württemberg, Germany) with a normal range of 0–7 mg/dL and an analytical sensitivity of 0.5 mg/dL. The transferases gamma-glutamyl transferase (GGT), alanine aminotransferase (ALT), and aspartate aminotransferase (AST) were measured using the COBAS INTEGRA 400 analyzer (Roche Diagnostic, Basel, Switzerland). Blood pressure was measured twice after a 10 min rest with a random zero mercury sphygmomanometer and then averaged following validated protocols [62].

### 2.5. Genotyping of the TNF-α -308 G/A

The genotyping of G308A promoter variant of the *TNF-α* gene was carried out as follows: Oligonucleotide primers and probes were designed with the Beacon Designer 4.0 (Premier Biosoft International, Palo Alto, CA, USA) as previously described [63]. The polymerase chain reaction (PCR) was carried out with 50 ng of genomic DNA from peripheral blood, 0.5 μL of each oligonucleotide primer (primer forward: 5′-CTG TCT GGA AGT TAG AAG GAAAC-3′; primer reverse: 5′-TGT GTG TAG GAC CCT GGA G-3′) and 0.25 μL of each probe (wild probe: 5′-Fam-AAC CCC GTC CTC ATG CCC-Tamra-3′; mutant probe: 5′-Hex-ACC CCG TCT TCA TGC CCC-Tamra-3′) with a final volume of 25 μL (Bio-Rad Laboratories, Hercules, CA, USA). DNA was denaturized at 95 °C for 3 min; this was followed by 50 cycles of denaturation at 95 °C for 15 s, and annealing at 59.3 °C for 45 s. The PCR was run on a final 25 μL reaction volume containing 12.5 μL of IQTM Supermix (Bio-Rad Laboratories, Hercules, CA, USA) with hot start Taq DNA polymerase.

Allelic discrimination was achieved using two sequence-specific probes labeled with distinct fluorescent dyes: one probe was labeled at the 5′ end with FAM and the other at the 3′ end with HEX. Each probe binds specifically to one allele of the *TNF-α* -308 G/A polymorphism. During amplification, the hybridization of the allele-specific probes to the target DNA allows the detection of fluorescence signals. FAM and HEX fluorescence were measured in real-time PCR to differentiate homozygous and heterozygous genotypes based on the presence of one or both signals [64]. Additionally, Hardy–Weinberg equilibrium was assessed to ensure the genotypic distribution in the study population.

### 2.6. Statistical Analyses

Quantitative variables were expressed as means ± standard deviations, whereas categorical variables were presented as percentages. Comparisons between subjects by BMI, CDAI (categorized), and genotypes were conducted using Student’s *t*-test, except for dietary variables, which were adjusted for total caloric intake and analyzed using an analysis of covariance (ANCOVA) and were presented as mean ± standard error of the mean.

To facilitate group comparisons, CDAI was dichotomized using its median value as the cut-off, due to the absence of established clinical thresholds. For BMI, we categorized participants based on WHO criteria, grouping those with class I obesity separately from those with class II and III obesity [56]. These categorizations were applied for *t*-test and ANCOVA analyses.

The χ^2^ test was used to calculate the Hardy–Weinberg equilibrium. Given the small number of individuals with the *TNF-α* -308 AA homozygous genotype, they were grouped with GA carriers under a dominant model. To assess the interaction between CDAI and TNF-α genotypes, two separate two-way factorial ANOVA models were conducted with insulin and HOMA-IR, respectively, as dependent variables. In each model, CDAI and TNF-α genotype were included as factors, and the interaction term (CDAI × genotype) was tested to evaluate effect modification. These models provided a formal test of interaction between dietary antioxidant intake and genotype in relation to insulin-related outcomes.

In addition, multivariate linear regression models were conducted to evaluate the independent associations of CDAI and genotype with insulin and HOMA-IR, including sex and BMI as covariates and were adjusted by energy intake. Beta coefficients and their corresponding 95% confidence intervals (CI) were reported as β (x; y). Age was not included due to collinearity with BMI and glucose-related variables.

Statistical tests were fitted in the statistical package IBM SPSS version 20.0 (IBM Inc., Armonk, NY, USA). The correlation plot was generated with Excel^®^ (version 2308, Microsoft Corp., Redmond, WA, USA). A *p*-value lower than 0.05 was established as statistically significant.

## 3. Results

A total of 292 Spanish adults with obesity were included in the analysis (mean age: 42.5 ± 13.2 years; 64% women). The overall distribution of TNF-α genotypes in the population was 73.8% for GG and 26.2% for GA/AA. When stratified by BMI category, genotype frequencies were nearly identical: in participants with BMI < 35 kg/m^2^, 73.7% had the GG genotype and 26.3% had GA/AA, while in those with BMI ≥ 35 kg/m^2^, 73.8% had the GG genotype and 26.2% had GA/AA (*p* = 0.977). Similarly, no significant differences in genotype distribution were observed when stratified by CDAI categories (*p* = 0.297). The TNF-α genotype distribution was consistent with Hardy–Weinberg equilibrium (*p* = 0.777).

Anthropometric, clinical, and biochemical parameters were examined across categories of BMI, CDAI, and TNF-α -308 G/A genotype (Table 1). As expected, individuals with a BMI ≥ 35 kg/m^2^ exhibited significantly higher values in most anthropometric parameters, systolic and diastolic blood pressure, insulin levels, HOMA-IR, TG, and TyG index compared to those with a BMI < 35 kg/m^2^ (*p* < 0.05).

Participants with a CDAI ≥ −0.3 arbitrary units (AU) showed significantly higher lean mass (*p* = 0.006). Regarding genotype, carriers of the A allele (GA/AA) displayed greater waist circumference (*p* = 0.048) and higher fat-to-lean mass ratio (*p* = 0.025) than those with the GG genotype (Table 1).

In terms of dietary intake, participants with higher CDAI values consumed significantly more energy (*p* < 0.001), carbohydrates (*p* = 0.006), and antioxidant nutrients, except for zinc (*p* = 0.059). Also, this group had a significantly lower lipid intake (*p* < 0.001) than those with lower CDAI values. No significant differences in dietary intake were observed by genotype or BMI (Table 2).

Multivariate regression models (Table 3) showed that higher CDAI was independently associated with lower insulin levels (β = −0.628, 95% CI: −0.976; −0.281, *p* < 0.001) and HOMA-IR (β = −0.169, 95% CI: −0.262; −0.075, *p* < 0.001), even after adjusting for sex, BMI, and genotype. The presence of the A allele was also independently associated with higher insulin (β = 2.347, 95% CI: 0.154; 4.539, *p* = 0.036) and HOMA-IR levels (β = 0.806, 95% CI: 0.144; 1.468, *p* = 0.017). Female sex was associated with lower insulin and HOMA-IR.

**Figure 1:**
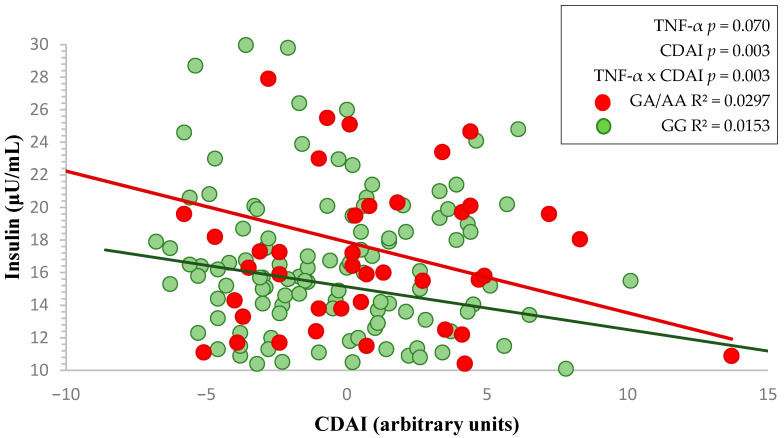
Two-way factorial ANOVA showing the interaction between *TNF-α* genotype and CDAI on fasting insulin levels. Red and green lines represent the linear regression slopes for GA/AA and GG genotypes, respectively.

The higher the CDAI, the lower the insulin values appear to be in these subjects (*p* = 0.151, Table 1). Moreover, subjects with the GA/AA genotypes showed higher, but not statistically significant, raw insulin levels compared to those with the GG genotype (*p* = 0.087, Table 1). Nevertheless, the higher the CDAI in participants with the TNF-α risk allele A, the more it contributed to reducing the slope of insulin levels compared to volunteers with the GG genotype (*p* = 0.070, Figure 1). This pattern was supported by a statistically significant interaction between CDAI and TNF-α genotype (*p* = 0.003, Figure 1), suggesting a potential effect modification associated with the genetic make-up.

**Figure 2:**
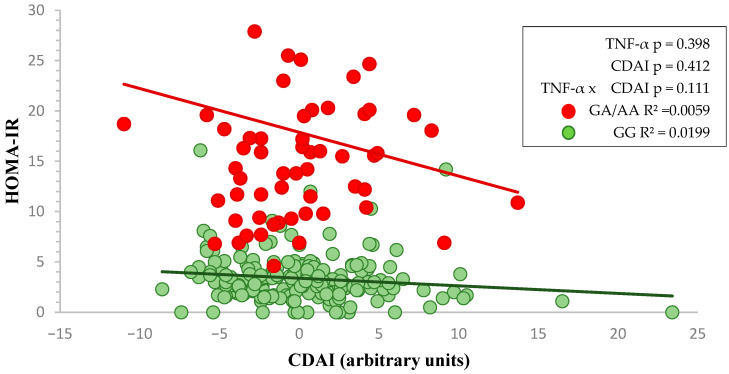
Two-way factorial ANOVA showing the interaction between *TNF-α* genotype and CDAI on HOMA-IR. Red and green lines represent the linear regression slopes for GA/AA and GG genotypes, respectively.

In a similar way, the interaction between HOMA-IR and CDAI related to TNF-α -308 rs1800629 genotypes (GG and GA/AA) is illustrated (Figure 2), although the slope is similar, the results are not statistically significant.

## 4. Discussion

This study provides novel insights into the relationship between the *TNF-α* -308 G/A polymorphism, dietary antioxidants, and nutrient–gene interactions in the regulation of insulin levels among Spanish adults with obesity. The findings suggest that higher dietary antioxidant intake is inversely associated with HOMA-IR and circulating insulin levels. Importantly, in our study, we observed a statistically significant interaction between *TNF-α* -308 G/A genotype and CDAI in relation to insulin levels, tested via a two-way ANOVA model. This suggests that the metabolic effect of dietary antioxidant intake may be modified by genetic background, particularly in individuals carrying the A allele. The data showed that as the CDAI increases in subjects with obesity carrying the *TNF-α* risk allele (A), a more pronounced downward trend in insulin levels was observed, compared to those with the GG genotype, supported by a statistically significant interaction.

This finding suggests that the interaction modifies the effect of the antioxidant on circulating insulin, depending on the genotype, since although circulating insulin levels decrease in subjects with the GG and AG/AA genotypes, the slope is steeper and the decrease in insulin levels is more evident in subjects with the risk genotype.

A high intake of antioxidant-rich foods has demonstrated protective effects against various metabolic diseases. Thus, in a large epidemiological study involving 21,831 healthy individuals, researchers used a semiquantitative food frequency questionnaire to assess diet and found a strong inverse association between plasma vitamin C levels and type 2 diabetes risk. To a lesser extent, fruit and vegetable intake were also associated with a significantly reduced type 2 diabetes risk [20]. Similarly, another study using the NHANES database has demonstrated an inverse relationship between CDAI and type 2 diabetes among 11,956 participants, independent of traditional risk factors. These findings further support the role of dietary antioxidants in preventing metabolic diseases [16]. Moreover, a study of Mexican children and adolescents highlighted that the dietary antioxidant index (DAI) is inversely associated with insulin resistance. Participants in the highest DAI category exhibited a significantly lower risk of insulin resistance (OR 0.49, 95% CI: 0.30; 0.80) compared to those in the lowest category. The association was particularly pronounced among women (OR 0.54, 95% CI: 0.29; 0.98) and individuals with excess weight (OR 0.37, 95% CI: 0.18; 0.76) [65]. However, when investigating the relationship between CDAI, calculated from 24-hour dietary recalls, insulin resistance and the TyG index in a cohort of 14,673 participants with a mean age of 50 years, the analysis revealed a significant inverse association between CDAI and TyG index, suggesting that increased antioxidant intake could mitigate this specific metabolic risk factor. Nevertheless, no direct link was identified between CDAI and insulin levels or insulin resistance [66]. According to these contradictory results, CDAI could influence some metabolic risk markers, but its role in insulin resistance is likely more complex. Further research is needed for a better understanding.

Nevertheless, the actual explanation could be that dietary antioxidants have been significantly associated with a reduction in ROS formation, a key element in the pathogenesis of insulin resistance [67]. ROS contribute to insulin resistance and impaired glucose metabolism by activating alternative signaling pathways that disrupt normal insulin function [6]. Additionally, antioxidants help mitigate lipid peroxidation, a process that generates harmful by-products leading to oxidative damage in cells, DNA, and proteins [68]. Lipid peroxidation, a hallmark of oxidative stress, results in cellular membrane damage, accelerated apoptosis, and cell death [42]. Finally, increased oxidative stress and lipid peroxidation are crucial elements of insulin resistance, β-cell dysfunction, glucose intolerance, and ultimately, type 2 diabetes mellitus [68].

In our results, fat mass/lean mass ratio, and waist circumference were higher in subjects with the GA/AA risk genotypes compared with the GG genotype, as observed in other studies [69,70,71]. However, other studies have reported conflicting results. For instance, research involving 194 Caucasian subjects found no significant differences in the -308 G/A allele frequency between lean and obese groups, nor associations with BMI, body fat distribution, insulin levels, or metabolic abnormalities, questioning the variant’s impact in this population [28]. De Luis et al. assessed 630 Spanish adults (75.5% GG genotype) and found no significant genotype-related differences in anthropometric, insulin resistance, lipid, or dietary intake variables [48]. Nevertheless, the same group demonstrated through nutritional interventions, that carriers of the risk allele presented more unfavorable metabolic responses [29,72]. However, possible ethnic differences in the effects of the *TNF-α* G-308A polymorphism were suggested, since in 440 Chinese subjects, there was no association with anthropometrics, insulin resistance, or lipid profile markers [73]. In contrast, a 2015 meta-analysis identified a significant association between this variant and elevated circulating insulin levels and HOMA-IR in Caucasian populations with obesity, suggesting a potential genetic predisposition to insulin resistance [30]. Thus, the tendency toward higher insulin levels in subjects with obesity carrying the A allele of the *TNF-α* gene, compared to those with the GG genotype, has not been universally observed.

The interplay between dietary antioxidants, the *TNF-α* -308 G/A polymorphism, and insulinemia is mediated by the connection between oxidative stress and inflammatory pathways [12]. In conditions of exacerbated inflammation, as observed in carriers of the A allele of the -308 polymorphism in the *TNF-α* gene, levels of reactive oxygen species (ROS) and oxidative stress are significantly elevated. Consequently, insulin concentrations, modulated by oxidative stress and inflammatory processes, are also higher. However, in our study, a greater attenuation of insulin levels was observed in subjects with the risk allele who had higher antioxidant consumption. In individuals suffering from acute inflammation, elevated ROS levels exceed the endogenous antioxidant defense capacity, leading to oxidative damage [74]. Dietary antioxidants can neutralize ROS, thereby mitigating oxidative damage, inhibiting nuclear factor kappa B (NF-κB) activation, and reducing the expression of pro-inflammatory cytokines, including TNF-α and IL-6 [75]. In contrast, individuals with lower inflammation levels, such as those with the non-risk genotype, experience minimal effects from dietary antioxidants due to the absence of substantial ROS accumulation. Furthermore, oxidative stress can be related to other genes, the inflammation process [76], and mitochondrial metabolic disorders [77]. Additionally, under conditions of pronounced oxidative stress, the demand for antioxidants is greater, reducing the likelihood of saturation and enhancing the efficacy of dietary antioxidant actions [78]. Dietary antioxidant consumption improves insulin signaling and nitric oxide production by reducing ROS-mediated interference, ultimately contributing to the attenuation of insulin levels in individuals with elevated inflammation [79]. Moreover, diet polyphenols can alleviate gut dysbiosis by scavenging ROS, increasing the abundance of the beneficial *Akkermansia muciniphila* bacteria (in obese mice, for example) and mitigate ROS production [39]. These assumptions may explain the observed impact in subjects with the risk allele and higher antioxidant intake. These findings are consistent with previous studies on gene–environment interactions. For example, Navas-Carretero et al. demonstrated that the impact of carbohydrate intake on glycaemia was modified by physical activity levels and genetic background in European adults, emphasizing the relevance of personalized dietary strategies based on genotype and lifestyle interactions [80].

Our findings support the growing body of evidence that antioxidant-rich diets may offer a practical intervention for those subjects with obesity and at genetic risk of insulin resistance, particularly in populations with a high prevalence of the *TNF-α* -308 G/A polymorphism, such as subjects with European ancestry [41]. The variability of results in other studies reinforces the significance of dietary antioxidants as a modifiable factor that could mitigate genetic predispositions.

This study has certain limitations that should be considered. The relatively small sample size limited subgroup analyses by homozygosity, sex, and age. Future studies should examine whether these factors modify the observed gene–diet associations, ideally leveraging larger datasets or advanced modeling techniques such as machine learning. As a cross-sectional design, causal relationships cannot be inferred—only associations. The use of self-reported dietary data and single insulin measurements may introduce variability. Data on physical activity were not available; however, energy intake (a surrogate marker in epidemiological research [81,82]) and BMI were included as adjustment variables to partly account for this. Evaluating only one genetic variant also limits broader genomic interpretations. The absence of TNF-α level measurements restricts biological plausibility assessments, and no renal function data (e.g., eGFR) were available, despite their relevance to insulin metabolism and antioxidant bioavailability. Nevertheless, this is, to our knowledge, the first study exploring the interaction between *TNF-α* rs1800629 and dietary antioxidants (via CDAI) in relation to insulinemia. Our findings are plausible and novel, and support future research on gene–nutrient interactions, especially involving other nutrients, gene–gene interactions, and diverse populations, considering that genetics, age, and environmental factors may affect generalizability.

These findings underscore the importance of tailoring dietary interventions to genetic backgrounds, highlighting the need to consider genetic variability in TNF-α expression, when assessing individual risk profiles for insulin disorders and designing personalized nutrition strategies. Although the genotype explained only approximately 3% of the variance in insulin levels, this result aligns with previous findings, indicating that common genetic variants account for around 3% of the heritability of BMI [83]. While modest, this proportion is meaningful in the context of genetic epidemiology, particularly considering the polygenic nature of insulin resistance and obesity, which show heritability estimates ranging from 20% to 70% [84,85]. However, a clear limitation would be the need for genotyping, which currently remains costly, but this challenge is expected to be overcome soon with advances in genotyping and bioinformatic tools that will facilitate pragmatic interpretations. Above all, the potential benefit of increasing antioxidant intake needs to be demonstrated in a randomized clinical trial, but the current findings can be translated into increase antioxidant consumption to implement nutrition advice with precision. Incorporating antioxidant-rich foods, such as fruits and vegetables, which are high in vitamins, minerals, and bioactive redox reduction nutrients, into the diet may serve as a non-pharmacological strategy to manage insulin resistance, particularly in individuals with inflammatory genotypes, such as *TNF-α* -308 G/A.

## 5. Conclusions

In summary, our results emphasize that dietary antioxidants may influence the relationship between *TNF-α* -308 G/A gene polymorphisms and insulin levels in individuals with obesity by mitigating oxidative stress, which may help reduce the effects on inflammatory pathways. This interaction highlights the potential of antioxidant-rich diets to alleviate insulin resistance, particularly in individuals with obesity carrying the genetic risk variant that increases TNF-α expression. Additionally, the interaction modifies the effect of antioxidants on insulin depending on the genotype. Although circulating insulin levels decrease in both GG and AG/AA genotype groups, the slope is steeper and the reduction in insulin levels is more evident in subjects with the risk genotype. These findings highlight the potential contribution of both genetic and dietary factors in shaping insulin levels within this population. While the data suggest that genetic background may modulate the relationship between antioxidant intake and glucose homeostasis in individuals with obesity, further research is needed to clarify the consistency and underlying mechanisms of this interaction.

## Figures and Tables

**Table 1:** Anthropometric, biochemical, and clinical characteristics of subjects with obesity according to BMI, CDAI, and *TNF-α* rs1800629 categories.

	BMI < 35 kg/m^2^ (*n* = 115)	BMI ≥ 35 kg/m^2^ (*n* = 173)	*p*	CDAI < −0.3 AU (*n* = 130)	CDAI ≥ −0.3 AU (*n* = 127)	*p*	GG Genotype (*n* = 214)	GA/AA Genotype (*n* = 76)	*p*
Women (%)	56.6	69.9	**0.024**	62.3	73.2	0.064	66.0	60.5	0.404
Weight (kg)	89.5 ± 11.5	107.5 ± 17.6	**<0.001**	99.0 ± 17.0	100.7 ± 17.8	0.426	99.8 ± 17.0	102.2 ± 19.9	0.315
BMI (kg/m^2^)	32.7 ± 1.5	40.8 ± 4.5	**<0.001**	37.5 ± 5.2	37.7 ± 5.3	0.751	37.5 ± 5.3	37.9 ± 5.6	0.527
Fat Mass (kg)	33.1 ± 8.7	47.5 ± 10.9	**<0.001**	43.2 ± 11.8	40.8 ± 12.5	0.145	41.0 ± 11.8	44.0 ± 13.5	0.111
Lean Mass (%)	61.9 ± 9.7	54.6 ± 7.2	**<0.001**	55.4 ± 7.9	58.9 ± 9.0	**0.003**	57.8 ± 9.0	56.6 ± 8.9	0.384
FM/LM Ratio	0.89 ± 0.12	1.05 ± 0.22	**<0.001**	0.98 ± 0.16	0.99 ± 0.23	0.951	0.97 ± 0.20	1.04 ± 0.20	**0.025**
Waist Circumference (cm)	103.8 ± 10.1	119.2 ± 11.9	**<0.001**	112.8 ± 13.8	113.8 ± 13.0	0.587	112.3 ± 13.4	116.0 ± 13.7	**0.048**
WHR	0.92 ± 0.08	0.95 ± 0.09	**0.020**	0.92 ± 0.09	0.95 ± 0.09	0.067	0.93 ± 0.09	0.95 ± 0.09	0.323
Glucose (mg/dL)	95.9 ± 19.3	97.4 ± 14.3	0.445	97.2 ± 15.9	96.3 ± 18.4	0.688	96.9 ± 17.4	96.4 ± 13.4	0.838
Insulin (μU/mL)	14.4 ± 10.7	18.3 ± 13.1	**0.007**	16.7 ± 9.9	15 ± 8.2	0.151	16.1 ± 11.8	19.0 ± 13.4	0.087
HOMA-IR	3.1 ± 2.9	4.3 ± 3.6	**0.002**	3.4 ± 3.2	3.3 ± 2.2	0.052	3.6 ± 3.3	4.3 ± 3.6	0.116
TC (mg/dL)	199.4 ± 37.1	196.5 ± 38.3	0.541	197.2 ± 41.1	196 ± 35.3	0.808	197.4 ± 37.8	197.5 ± 38.3	0.989
LDL-c (mg/dL)	125.2 ± 33.1	121.6 ± 33.9	0.386	122.9 ± 36.5	121.2 ± 31.4	0.700	123.4 ± 33.7	121.3 ± 33.8	0.643
HDL-c (mg/dL)	50.7 ± 13.8	48.6 ± 12.5	0.195	50.5 ± 13.4	49.3 ± 13.3	0.512	49.4 ± 13	49.3 ± 13.1	0.932
TG (mg/dL)	111.3 ± 53.5	134.2 ± 75.3	**0.003**	119.8 ± 57.3	127.9 ± 79.0	0.357	122.9 ± 61.3	130.9 ± 85.2	0.387
TyG index	10.5 ± 1.2	10.9 ± 1.2	**0.002**	10.7 ± 1.11	10.8 ± 1.3	0.660	10.7 ± 1.2	10.8 ± 1.2	0.666
AST (U/L)	22.3 ± 8.8	22.8 ± 10.4	0.702	21.8 ± 7.7	23.2 ± 10.7	0.249	22.5 ± 9.5	22.8 ± 10.5	0.842
ALT (U/L)	30.4 ± 13.5	34.4 ± 22.2	0.074	31.6 ± 18.2	34.5 ± 20.2	0.236	32.7 ± 18.6	33.6 ± 21.6	0.723
GGT (U/L)	29.1 ± 16.6	30.4 ± 23.3	0.630	27.1 ± 16.8	31.2 ± 21.7	0.209	29.2 ± 21.8	31.6 ± 17.6	0.416
CRP (mg/dL)	3.6 ± 5.5	5.4 ± 12.0	0.266	5.9 ± 14.1	3.6 ± 5.0	0.177	3.8 ± 6.0	7.5 ± 16.8	0.151
SBP (mmHg)	122.1 ± 14.5	132.6 ± 16.9	**<0.001**	130.3 ± 16.4	127.2 ± 17.4	0.188	129.1 ± 17.5	127.1 ± 14.7	0.397
DBP (mmHg)	76.9 ± 10.9	83.9 ± 12	**<0.001**	82.5 ± 11.4	79.8 ± 12.9	0.101	81.7 ± 12.0	79.5 ± 12.3	0.196

All data are presented as mean ± standard deviation. *p* values were derived using Student’s *t*-test. Bold numbers indicate *p* < 0.05. Discrepancies in the total number of subjects are due to missing values in the main variables. ALT: alanine aminotransferase; AST: aspartate aminotransferase; AU: arbitrary units; BMI: body mass index; CRP: c-reactive protein; DBP: diastolic blood pressure; FM/LM: fat mass/lean mass; GGT: gamma-glutamyl transferase; HC: hip circumference; HDL-c: high-density lipoprotein cholesterol; HOMA-IR: homeostasis model assessment; LDL-c: low-density lipoprotein cholesterol; SBP: systolic blood pressure TC: total cholesterol; TG: triglycerides; TyG: triglyceride–glucose; WHR: waist-to-hip ratio.

**Table 2:** Dietary characteristics (mean daily intake) of subjects with obesity according to BMI, CDAI, and *TNF-α* rs1800629 categories.

	BMI < 35 kg/m^2^ (*n* = 115)	BMI ≥ 35 kg/m^2^ (*n* = 173)	*p*	CDAI < −0.3 AU (*n* = 130)	CDAI ≥ −0.3 AU (*n* = 127)	*p*	GG Genotype (*n* = 214)	GA/AA Genotype (*n* = 76)	*p*
Energy (Kcal)	1956 ± 733	2033± 787	0.438	1643 ± 484	2353 ± 828	**<0.001**	2012 ± 814	2001 ± 612	0.916
Protein (% energy)	19.5 ± 0.5	19.6 ± 0.4	0.905	19.3 ± 0.5	19.7 ± 0.5	0.579	19.6 ± 0.4	19.2 ± 0.7	0.558
Lipids (% energy)	41.3 ± 0.9	40.7 ± 0.7	0.649	44.1 ± 0.9	38.4 ± 0.8	**<0.001**	40.9 ± 0.7	40.9 ± 1.1	0.960
Carbohydrate (% energy)	39.4 ± 1.0	39.7 ± 0.8	0.832	36.9 ± 1.0	41.8 ± 0.9	**<0.001**	39.5 ± 0.7	39.9 ± 1.3	0.779
Fiber (g)	16.4 ± 0.6	15.2 ± 0.5	0.143	11.9 ± 0.5	19.3 ± 0.5	**<0.001**	15.6 ± 0.5	16 ± 0.8	0.664
Vitamin A (μg)	1617.2 ± 109.9	1398.2 ± 86.5	0.119	1027.0 ± 96.7	1923.1 ± 95.0	**<0.001**	1531.0 ± 79.5	1340.1 ± 135.5	0.226
Vitamin C (mg)	138.0 ± 11.0	145.4 ± 8.7	0.595	97.7 ± 9.7	186.1 ± 9.5	**<0.001**	146.6 ± 8.0	132.3 ± 13.6	0.366
Vitamin E (mg)	8.9 ± 0.4	8.0 ± 0.3	0.076	7.1 ± 0.4	9.5 ± 0.4	**<0.001**	8.3 ± 0.3	8.4 ± 0.5	0.946
Copper (μg)	1109.0 ± 55.7	979.9 ± 43.9	0.070	797.9 ± 49.1	1253.6 ± 48.2	**<0.001**	1055.4 ± 40.4	964.0 ± 68.9	0.254
Selenium (μg)	74.7 ± 3.3	73.7 ± 2.6	0.819	63.9 ± 3	84.0 ± 3.0	**<0.001**	74.0 ± 2.4	75.3 ± 4.1	0.789
Zinc (mg)	13.2 ± 7.6	23.1 ± 6.0	0.310	9.1 ± 7.1	29.2 ± 7.1	0.059	16.6 ± 5.5	27.5 ± 9.4	0.317
Omega-3 (g)	0.38 ± 0.07	0.45 ± 0.06	0.481	0.21 ± 0.07	0.63 ± 0.07	**<0.001**	0.45 ± 0.05	0.34 ± 0.09	0.313
CDAI (AU)	0.08 ± 4.0	−0.07 ± 4.4	0.778	−3.26 ± 1.83	3.16 ± 3.49	**<0.001**	0.15 ± 4.34	−0.39 ± 4.05	0.370

All nutrients are presented as mean ± SEM. *p* values were derived using ANCOVA adjusted by energy, except for energy and CDAI variables. Bold numbers indicate *p* < 0.05. Discrepancies in the total number of subjects are due to missing values in the main variables. AU: arbitrary units; BMI: body mass index; CDAI: Composite Dietary Antioxidant Index; Kcal: kilocalories. CDAI categorized by median cut-off (−0.3 AU); last row shows CDAI as continuous variable (mean ± SE).

**Table 3:** Multivariate analysis of parameters associated with insulin levels and HOMA-IR as dependent variables in subjects with obesity.

Insulin	β (95% CI)	*p*	*R* ^2^
Sex	−3.235 (−5.678; −0.793)	0.010	0.014
BMI (kg/m^2^)	0.428 (0.223; 0.633)	<0.001	0.101
CDAI (AU)	−0.628 (−0.976; −0.281)	<0.001	0.020
*TNF-α* -308 G/A genotypes	2.347 (0.154; 4.539)	0.036	0.012
Total *R*^2^			0.152
**HOMA-IR**	**β (95% CI)**	***p***	***R*** **^2^**
Sex	−0.955 (−1.683; −0.226)	0.010	0.024
BMI (kg/m^2^)	0.108 (0.046; 0.170)	0.001	0.105
CDAI (AU)	−0.169 (−0.262; −0.075)	<0.001	0.015
*TNF-α* -308 G/A genotypes	0.806 (0.144; 1.468)	0.017	0.009
Total *R*^2^			0.137

Multiple linear regression models for insulin (µU/mL) and HOMA-IR as dependent variables were adjusted for energy (Kcal/day). AU: arbitrary units; β: beta-coefficient; BMI: body mass index; CDAI: Composite Dietary Antioxidant Index; CI: confidence interval. Note: All models were adjusted for total daily energy intake (Kcal), although energy was not included as a reported variable in the table.

## Data Availability

The data that support the findings of this study are available on request from the corresponding author due to the use of health data with high protection levels, according to the current regulations in our country.

## Abbreviations

The following abbreviations are used in this manuscript:

AMPK	AMP-activated protein kinase
ANCOVA	analysis of covariance
ALT	alanine aminotransferase
AST	aspartate aminotransferase
AU	arbitrary units
BMI	body mass index
CI	confidence interval
CDAI	Composite Dietary Antioxidant Index
CRP	C-reactive protein
DBP	diastolic blood pressure
FABP	fatty acid-binding protein
FM/LM	fat mass/lean mass
GGT	gamma-glutamyl transferase
HC	hip circumference
HOMA-IR	homeostatic model assessment for insulin resistance
IL-6	interleukin 6
IRS-1	insulin receptor substrate-1
KCNJ11	potassium channel, inwardly rectifying subfamily J member 11
Kcal	kilocalories
LDL-C	low-density lipoprotein cholesterol
NF-κB	nuclear factor kappa B
PCR	polymerase chain reaction
PPAR-γ	peroxisome proliferator-activated receptor gamma
ROS	reactive oxygen species
RT-PCR	Real-time polymerase chain reaction
SBP	systolic blood pressure
SNV	single nucleotide variants
TC	total cholesterol
TG	triglycerides
TNF-α	tumor necrosis factor-alpha
TyG	TG and glucose index
WHR	Waist-to-hip ratio
